# Comparative transcriptome findings reveal the neuroinflammatory network and potential biomarkers to early detection of ischemic stroke

**DOI:** 10.1186/s13036-023-00362-8

**Published:** 2023-08-02

**Authors:** Jiefeng Luo, Dingzhi Chen, Yujia Mei, Hepeng Li, Biyun Qin, Xiao Lin, Ting Fung Chan, Keng Po Lai, Deyan Kong

**Affiliations:** 1 Department of Neurology, The Second Affiliated Hospital of Guangxi Medical University, No 166 Dadaxuedong Road, Nanning, Guangxi 530007 People’s Republic of China; 2 Department of Psychiatry, Icahn School of Medicine at Mount Sinai, New York, USA; 3 School of Life Sciences, State Key Laboratory of Agrobiotechnology, The Chinese University of Hong Kong, Hong Kong SAR, China; 4 Clinical Medicine Research Center, The Second Affiliated Hospital of Guangxi Medical University, No 166 Dadaxuedong Road, Nanning, Guangxi 530007 P. R. China

**Keywords:** Neuroinflammation, Biomarkers, Ischemic stroke, Therapeutic targets, Transcriptome analysis

## Abstract

**Introduction:**

Ischemic stroke accounts for 70–80% of all stroke cases, leading to over two million people dying every year. Poor diagnosis and late detection are the major causes of the high death and disability rate.

**Methods:**

In the present study, we used the middle cerebral artery occlusion (MCAO) rat model and applied comparative transcriptomic analysis, followed by a systematic advanced bioinformatic analysis, including gene ontology enrichment analysis and Ingenuity Pathway Analysis (IPA). We aimed to identify novel biomarkers for the early detection of ischemic stroke. In addition, we aimed to delineate the molecular mechanisms underlying the development of ischemic stroke, in which we hoped to identify novel therapeutic targets for treating ischemic stroke.

**Results:**

In the comparative transcriptomic analysis, we identified 2657 differentially expressed genes (DEGs) in the brain tissue of the MCAO model. The gene enrichment analysis highlighted the importance of these DEGs in oxygen regulation, neural functions, and inflammatory and immune responses. We identified the elevation of angiopoietin-2 and leptin receptor as potential novel biomarkers for early detection of ischemic stroke. Furthermore, the result of IPA suggested targeting the inflammasome pathway, integrin-linked kinase signaling pathway, and Th1 signaling pathway for treating ischemic stroke.

**Conclusion:**

The results of the present study provide novel insight into the biomarkers and therapeutic targets as potential treatments of ischemic stroke.

**Supplementary Information:**

The online version contains supplementary material available at 10.1186/s13036-023-00362-8.

## Background

Stroke is the second-leading cause of death and the most frequent cause of permanent disability in adults worldwide [1, 2]. Strokes are classified as either ischemic or hemorrhagic. Ischemic stroke accounts for 70–80% of the cases [3]. According to the data from the World Stroke Organization (https://www.world-stroke.org), there are over 9.5 million new cases of ischemic stroke, leading to over 2.7 million deaths per year globally. Due to the changes in living habits, stroke also tends to occur at a younger age [4]. Hence, stroke remains one of the major health care problems with high death and disability rates. There are many risk factors of stroke, including high body mass index, high fasting plasma glucose, high low-density lipoprotein cholesterol, high red meat diet, alcohol consumption, and second-hand smoking [1]. In a molecular pathological study of ischemic stroke, cumulating evidence suggested that ischemic injury and inflammation account for its pathogenic progression [5]. Additionally, the inflammatory response is one of the causes of brain damage in cerebral ischemia [3]. Furthermore, a randomized controlled study of ischemic stroke patients demonstrated that tumor necrosis factor-α and the interleukins (IL), such as IL-1β, IL-6, and IL-20, were associated with inflammation in ischemic stroke [6]. More interestingly, depletion of regulatory T lymphocytes significantly delayed brain damage and deteriorated functional outcomes in acute experimental stroke [7]. Hence, a better understanding of the molecular pathogenesis and inflammatory pathways of ischemic stroke may provide information related to diagnostic, prognostic, and therapeutic markers to tackle ischemic stroke.

In the present study, we used the middle cerebral artery occlusion (MCAO) mouse model and applied comparative transcriptome sequencing, followed by systematic bioinformatic analysis, including gene ontology (GO) and Ingenuity Pathway Analysis (IPA) to delineate the molecular mechanisms underlying the development of ischemic stroke. Our result showed the differential gene expression in the brain tissue of the MCAO model. The result of bioinformatic analysis further highlighted the importance of these genes in neuroinflammation and deteriorated neurological functions. More importantly, we have identified some novel biomarkers, including angiopoietin-2 (Angpt2) and leptin receptor (Lepr), for the detection of ischemic stroke. Angpt2 is reported to play a role in vascular physiology and pathophysiology [8]. Lepr is found to be expressed in different types of vascular lesions [9]. The present findings provide a novel set of biomarkers that can be used for the early detection of ischemic stroke and can be used for therapeutic interventions.

## Material and methods

### Animal maintenance

Male Sprague Dawley rats (240 ± 20 g, Specific Pathogen Free, 9 weeks old) were obtained from the central animal facility of Guangxi Medical University (Nanning, China). The animals were housed under standard conditions of light and dark cycles (12 h:12 h; temperature, 25 °C) with free access to food and water. Each rat was housed in a separate cage to avoid interference in the housing environment. In addition, the cages were regularly cleaned. All the animal studies were conducted according to the approved protocols and guidelines of the Institutional Animal Ethical Care Committee of Guangxi Medical University Experimental Animal Center.

### Cerebral ischemia model establishment

The establishment of the cerebral ischemia model has been described in a previous article [10]. Seven rats were used per group. Briefly, the rats were anesthetized with chloral hydrate (10%, 3 mL/kg); the inner and outer muscles of the sternocleidomastoid muscle were separated to expose and isolate the right common, external, and internal carotid arteries. The model was established by inserting a monofilament (approximately 2 cm) from the external carotid artery to the middle cerebral artery, avoiding the pterygopalatine artery. After the monofilament was inserted, the common carotid artery was ligated to complete the ipsilateral ischemia. After 2 h of ischemia, the monofilament was gently pulled out, and the ligation of the common carotid artery was relieved to form reperfusion. The wound was disinfected with iodine and sutured. The MCAO model was confirmed by three monitors. Laser-Doppler flowmetry (LDF) guided fiber insertion was used to monitor the ipsilateral blood flow, modified neurological severity score (mNSS) was used to evaluate overall neurological function, and triphenyl tetrazolium chloride staining was used to evaluate infarct size.

### Neurological deficit evaluation

The modified neurologic severity score (mNSS) was used to evaluate the neurobehavioral outcome 1 day after MCAO as described in a previous study [11]. There are four tests in the scoring systems, including motor, sensory, balance, and reflex tests. Scores from all the tests were summed, where 0 represents no deficit and 18 represents maximal deficit.

### Infarct volume assessment

At 24 h after the reperfusion, rats were euthanized by intraperitoneal injection of a 1% solution of pentobarbital sodium at a dose of 40 mg/kg. Then, ophthalmic scissors and surgical forceps were used to adequately expose the chest and abdomen. After clamping the abdominal aorta, an injection needle was inserted into the apex of the heart, followed by incising the right atrium to allow venous blood to drain. Approximately, 100 mL of physiological saline solution was injected through the injection needle to ensure successful perfusion, indicated by the whitening of the lungs and liver in the rat. Subsequently, brain tissue sampling was conducted. Brains were immediately removed and cut into five serials of 2 mm-thick coronal slices. A 2% solution of 2,3,5-triphenyl tetrazolium chloride was used to assess the infarct zone (white zone). Image-J® (image-processing software) was used by an independent observer, who was blinded to the group status, to measure the ischemic area (the unstained areas) and to calculate the infarct volume in each mouse brain. The infarct area was calculated as the area of the non-ischemic hemisphere minus the non-infarcted area of the ischemic hemisphere. Infarct volume = infarct area × thickness (2 mm). The percent of cerebral infarction was calculated using the following formula: The percentage of cerebral infarction = infarct volume /the volume of the non-ischemic hemisphere × 100.

### Transcriptome sequencing and bioinformatic analysis

After the rats were sacrificed (five rats per condition), the cerebral cortex ischemic penumbra was isolated and immediately placed in liquid nitrogen cryopreservation until sequencing. The cerebral ischemic core area of MCAO rats was white, and the brain tissue around the ischemic penumbra was regarded as the ischemic core area, which is intuitive. The ischemic penumbra of the cerebral cortex with a width of 1 mm around the white cerebral ischemic core area was used for sequencing. Total RNA was extracted using Trizol reagent following the manufacturer's instruction as previously described [12]. The total RNA quantity and purity were determined using Bioanalyzer 2100 and RNA 6000 Nano LabChip Kit (Agilent, CA, USA, 5067–1511). High-quality RNA samples (five replicates per condition), with an RNA integrity number > 7.0, were used to construct the sequencing library previously described [12]. The average insert size for the final cDNA library was 300 ± 50 bp. The 2 × 150 bp paired-end sequencing (PE150) was performed using the DNBSEQ sequencer following the vendor's recommended protocol. Reads obtained from the sequencing were further filtered using Cutadapt to remove the low-quality reads (https://cutadapt.readthedocs.io/en/stable/, version:cutadapt-1.9). The clean reads were aligned to the rat reference genome (rn6) using the HISAT2 (https://daehwankimlab.github.io/hisat2/, version:hisat2-2.0.4) package. The mapped reads of each sample were assembled using StringTie (http://ccb.jhu.edu/software/stringtie/, version:stringtie-1.3.4d) with default parameters. Then, all transcriptomes from all samples were merged to reconstruct a comprehensive transcriptome using gffcompare software (http://ccb.jhu.edu/software/stringtie/gffcompare.shtml, version:gffcompare-0.9.8). After the final transcriptome was generated, StringTie and ballgown (http://www.bioconductor.org/packages/release/bioc/html/ballgown.html) were used to estimate the expression levels of all transcripts and perform expression abundance for mRNAs by calculating fragment per kilobase of transcript per million. Differential gene expression analysis was performed using DESeq2 software. The genes with the parameter of false discovery rate below 0.05 and fold change ≥ 2 were considered differentially expressed genes (DEGs). DEGs were then subjected to GO enrichment analysis and Ingenuity Pathway Analysis (IPA). The Z-score is determined by using the IPA. The DEGs of each treatment group were uploaded to the IPA system for functional analysis (QIAGEN, Hilden, Germany). Canonical Pathway Analysis of IPA was used to identify significantly enriched canonical pathways in each treatment group, and Diseases and Functions Analysis of IPA was used to identify significantly enriched diseases and biological functions in each treatment group. The pathway or function with *P*-value < 0.05 was considered statistically significant. The activation z‐score is to infer the activation states of predicted transcriptional regulators. The activation state of an upstream regulator is determined by the regulation direction associated with the relationship from the regulator to the gene.

### Immunohistochemistry staining

The brain tissue section was blocked with bovine serum albumin solution (5%, v/v) for 1 h at room temperature. Then the section was incubated with primary antibodies against ANGPT2 or LEPR (1:200) at 4℃ overnight. The secondary antibody with horseradish peroxidase was used to bind the antigen antibody complex. The positive labeled cells were counted under microscopy system.

## Results

### Neurologic deficits in the MCAO rat model

**Fig. 1:**
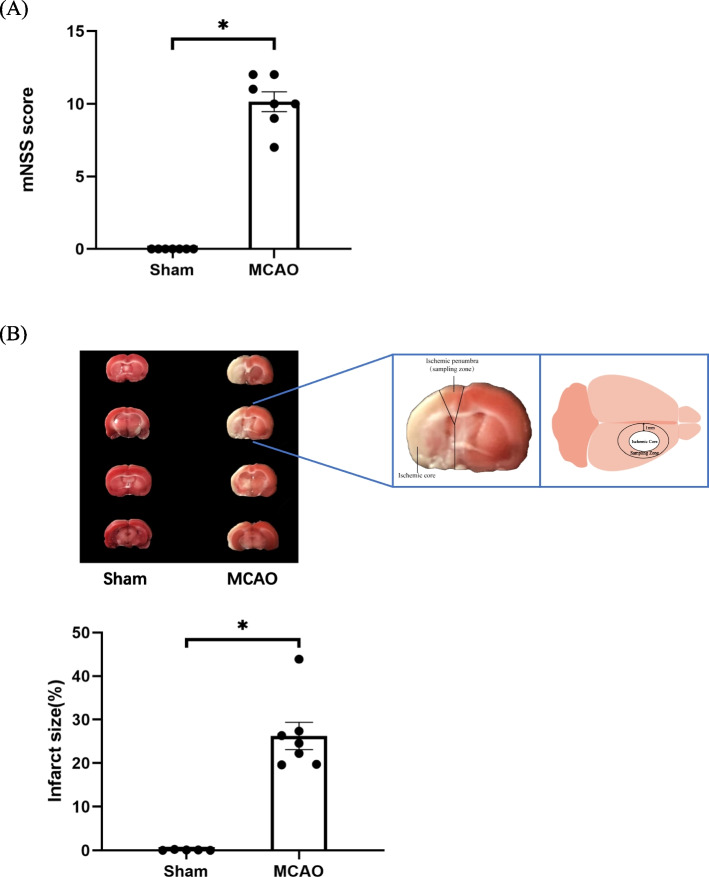
Neurologic deficits in the middle cerebral artery occlusion (MCAO) rat model. **A** The modified neurologic severity score and **B** the infarct volume were used to evaluate the neuro-clinicopathological deficits in the MCAO model. The picture showed the ischemic penumbra of the cerebral cortex harvested for sequencing analysis. *N* = 7; the data are shown as mean ± SD

To investigate the biomarkers and molecular mechanisms underlying the development of stroke, the MCAO model was established. The neurobehavioral outcome was evaluated by using the mNSS, including motor, sensory, balance, and reflex tests, after MCAO. We found that the mNSS score was 10 in the MCAO group (Fig. 1A) (*p* < 0.05), suggesting the severe neurological deficits in the ischemic stroke models. In addition, we found a significant induction of infarct volume, which reflected the clinicopathological deficits in the brain caused by an ischemic stroke (Fig. 1B) (*p* < 0.05).

### Alteration of biological functions led to brain disorders in the MCAO model

**Fig. 2:**
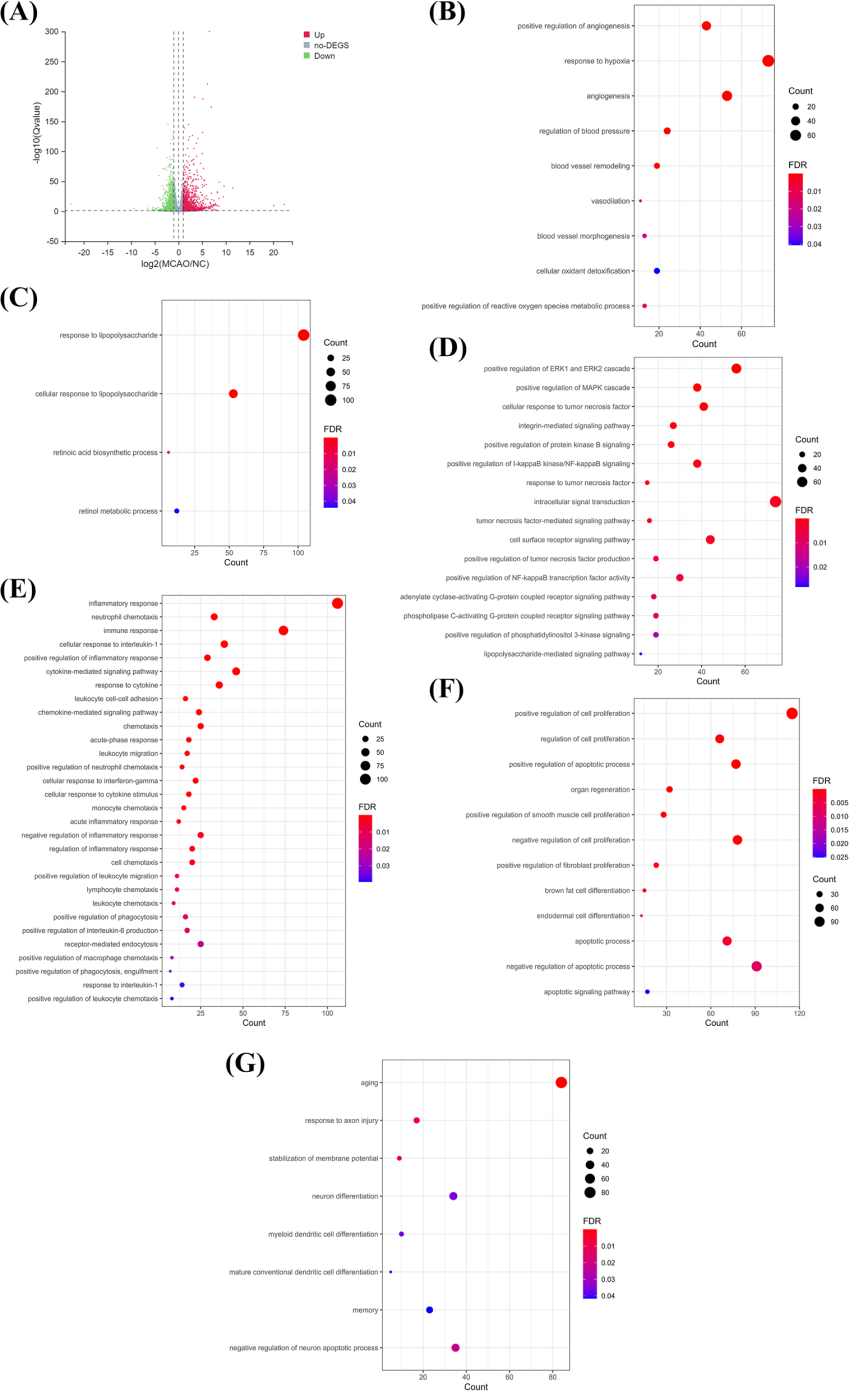
Differentially expressed genes are involved in biological processes related to oxygen regulation, metabolism, cell signaling, and inflammation in the middle cerebral artery occlusion (MCAO) model. **A** The volcano plot showed the differential gene expression in the brain tissue of the MCAO model. The X-axis represents the fold change of the difference MCAO and control group after conversion to log 2, and the Y-axis represents the significance q-value after conversion to -log 10. Red dots represent upregulated genes, green dots represent downregulated genes, and grey dots represent non-changed genes. Gene ontology enrichment analysis highlighted the involvement of the differentially expressed genes in **B** oxygen regulation, **C** metabolism, **D** cell signaling, **E** inflammatory and immune responses, **F** cell proliferation, and apoptosis, and **G** neurological functions. The size of the dots represents the number of genes, and the color of the dots represents the significance of the processes

To investigate the DEGs in the stroke model, a comparative transcriptomic analysis was conducted. We obtained at least 43 million clean reads per sample and translated a total of 66.15 Gb of clean data. By comparing the gene expression profile between the MCAO group and the control group, we identified 2657 DEGs, including 1517 upregulated genes and 1140 downregulated genes (Fig. 2 & Additional file 1) (q < 0.05). Then, the DEGs were subjected to GO enrichment analysis to determine the alteration of biological processes in the stroke model. In the analysis, we mainly focused on the processes and signaling related to the development and adverse outcomes of stroke. The results of GO highlighted many biological processes related to oxygen regulation, including regulation of angiogenesis, regulation of reactive oxygen species, response to hypoxia, regulation of blood pressure, blood vessel remodeling, vasodilation, blood vessel morphogenesis, and cellular oxidant detoxification (Fig. 2B & Table 1) (*p* < 0.05). The dysregulation of oxygen could lead to alteration of metabolic processes, including the response to the lipopolysaccharide and retinoic acid biosynthetic process, retinol metabolic process (Fig. 2C & Table 2) (*p* < 0.05), and alteration of cell signaling pathways, such as one involved in the regulation of the extracellular signal-regulated kinase 1 and extracellular signal-regulated kinase 2 cascades, the mitogen-activated protein kinase cascade, protein kinase B signaling, I-kappaB kinase/NF-kappaB signaling, tumor necrosis factor production, NF-kappaB transcription factor activity, phosphatidylinositol 3-kinase signaling, and lipopolysaccharide-mediated signaling pathway in the brain tissue of the stroke model (Fig. 2D and Table 3) (*p* < 0.05). These alterations might result in immune and inflammatory responses (Fig. 2E and Table 4) (*p* < 0.05) and alteration of cell proliferation and cell apoptosis (Fig. 2F and Table 5) (*p* < 0.05). The outcomes would be neurological disorders and diseases such as aging, axon injury, disruption of membrane potential, neuron differentiation, myeloid dendritic cell differentiation, and memory (Fig. 2G and Table 6) (*p* < 0.05).

**Table 1:** Alteration of biological functions related to angiogenesis and hypoxia in MACO model

Biological process	Number of gene	FDR	Genes
Positive regulation of angiogenesis	43	8.11E-09	ECM1, ITGB3, C5AR1, SERPINE1, ITGB2, HSPB1, ADM, FGF1, THBS1, ETS1, FGF2, HIF1A, CX3CL1, AQP1, C3, LGALS3, CYSLTR2, C6, CXCR2, ZC3H12A, C3AR1, HMOX1, CYP1B1, CD34, ANGPT2, PTGIS, ANXA3, NOS3, SPHK1, CYBB, CELA1, MMP9, F3, TNFRSF1A, RUNX1, EREG, LRG1, DDAH1, IL1B, ADM2, CHI3L1, RAPGEF3, ENG
Response to hypoxia	73	1.08E-08	SERPINA1, HP, ADM, PLAT, PLOD2, PYGM, ECE1, HIF3A, TRH, TNF, ETS1, CX3CL1, LOXL2, SLC6A4, ICAM1, NPPB, NPPC, EDNRA, ALKBH5, PLAU, MB, CASP3, LEPR, PTGIS, ITGA2, MMP3, PRKCD, NGB, DIO3, CYBA, MMP9, HSPG2, TNFRSF1A, F7, MMP14, MMP13, CLDN3, IL1B, ALDOC, ANGPTL4, CRYAB, ENG, SHC1, CXCR4, HIF1A, HSPD1, PDLIM1, SOCS3, LDHA, MUC1, HMOX1, CCL2, ABCB1A, CAMK2G, LDLR, TGFB2, VCAM1, ANGPT2, TGFB1, ACE, NOS2, NOS3, TGFB3, CFLAR, IGF1, MT3, EPOR, FOSL2, CXCL12, GPR182, TACC3, FAS, ADA
Angiogenesis	53	3.55E-07	CLIC4, ECM1, NRP2, PLXND1, SERPINE1, HIF3A, FGF1, RBPJ, FGF2, TMEM100, ESM1, CASP8, SOX17, PLAU, GJA5, ANPEP, ZC3H12A, LEPR, RSPO3, PROK2, CYP1B1, EPHB3, ANXA2, MYH9L1, EREG, MMP14, ANGPTL4, MFGE8, RAPGEF3, ENG, CCL12, SHC1, THY1, PTGS2, HIF1A, DLL4, SRPX2, S1PR1, HMOX1, FLNA, TBX1, JUN, ANGPT2, NOS3, WNT7B, MCAM, FN1, TBX4, ADM2, FGF18, TGFBI, PLCD1, FGFR1
Regulation of blood pressure	24	1.63E-05	CALCA, ACE, NOS2, GCH1, ATP1A2, CYBA, ECE1, TACR1, ATP1A1, PTGS2, TRHDE, AGT, AGTR1A, PTGS1, SULT1A1, EDNRA, TRPV4, NPY, SCNN1A, C3AR1, HMOX1, PPARG, CD34, MYH6
Blood vessel remodeling	19	1.34E-04	TBX1, TGFB2, NOS2, SEMA3C, NOS3, ELN, CHD7, LIF, BGN, IGF1, AHR, RBPJ, DLL4, CSRP3, CBS, FLNA, RSPO3, BAK1, TGM2
Vasodilation	11	0.019	CALCA, CASR, GCH1, ALB, ITGA1, BDKRB2, APOE, KNG2, AGT, KNG1, CFTR
Blood vessel morphogenesis	13	0.019	TBX1, ANGPT2, FLT4, ETV2, AHR, SHB, THBS1, HIF1A, AMOT, GJA1, COL4A1, CYP1B1, FGFR1
Cellular oxidant detoxification	19	0.041	GPX2, SRXN1, TXNRD1, HP, MGST2, GSTT1, LTC4S, PTGS2, PRDX6, PTGS1, GSTZ1, DUOX1, SESN2, PXDN, APOE, GSTM7, S100A9, DUOX2, S100A8
Positive regulation of reactive oxygen species metabolic process	13	0.010	CDKN1A, RIPK3, GADD45A, ACOD1, F2, THBS1, PLAU, DUOXA1, ZC3H12A, TSPO, CYP1B1, CD36, TP53

**Table 2:** Alteration of biological functions related to metabolism in MACO model

Biological process	Number of gene	FDR	Genes
Response to lipopolysaccharide	104	9.23E-24	CD86, IL1RN, NCF2, PROS1, SERPINE1, HP, TNF, ICAM1, NPPB, EDNRA, LGALS9, GGT1, IL6R, SLC11A1, DIO2, ACOD1, TICAM1, MAPKAPK3, SLPI, TRIB1, S100A9, S100A8, ABCG2, PTGES, PTGFR, MAOB, CNP, C5AR1, MGST2, CSF2RB, TNFRSF11B, MEFV, CYP27B1, SOCS3, SOCS1, THPO, CD14, NGFR, JUN, VCAM1, ACE, TNFRSF10B, IGF1, SERPINA3N, NFKB2, SELP, CXCL10, CXCL11, IL6, TG, GJB6, TRPV4, CD27, MYD88, PCSK1, CXCL6, CD40, CXCL9, SERPINA1, HMGB2, CXCL1, ADM, CXCL3, LITAF, THBD, CASP8, CASP3, ACP5, CCR7, LBP, JAK2, TIMP4, ABCC3, PTGIR, MMP3, PAWR, PLA2G4A, TNFRSF1B, NGF, MMP9, F3, TNFRSF1A, IL1B, TNFRSF26, CEBPB, PTGER2, PTGER3, NOD2, PTGS2, LTC4S, HSPD1, MUC2, CNR2, NOCT, CCL2, RELT, NOS2, GCH1, NOS3, ERBIN, FMO1, FAS, LTBR
Cellular response to lipopolysaccharide	53	1.21E-08	CD86, CXCL6, CSF3, CD40, SERPINE1, HMGB2, CXCL3, TNF, LITAF, CXCL2, CXCL16, ICAM1, PYCARD, FCGR3A, ZFP36, PLAU, ZC3H12A, PDE4B, TSPO, CD36, HMGCS2, LBP, JAK2, MAP2K3, ACOD1, TICAM1, TNFRSF1B, MMP9, PLSCR1, IL1B, RARA, IRF8, CEBPB, PTGER3, LTC4S, HIF1A, SBNO2, ADAMTS13, THPO, P2RY2, NLRP3, CCL2, CD14, GBP2, NOS2, CCL20, FN1, CXCL10, IL6, TNIP2, LCN2, FCGR2B
Retinoic acid biosynthetic process	6	0.022	ALDH1A3, DHRS9, CRABP2, RDH10, RBP1, ALDH1A1
Retinol metabolic process	12	0.044	ALDH1A3, RBP4, AKR1B10, RDH10, RBP1, RDH7, ALDH1A1, CYP1B1, LRAT, PLB1, RPE65, AKR1B8

**Table 3:** Alteration of biological functions related to cell signaling in MACO model

Biological process	Number of gene	FDR	Genes
Positive regulation of ERK1 and ERK2 cascade	56	2.62E-08	ARHGAP8, FLT4, ITGB3, HTR2B, HTR2C, FGF2, CX3CL1, ICAM1, PYCARD, C1QTNF3, EDNRA, CYSLTR2, GLIPR2, CD36, LGALS9, NPY5R, DCC, IL1B, RARA, CHI3L1, CD44, CCL12, C5AR2, C5AR1, NOD2, C3, CCL9, THPO, GPNMB, CCL7, CCL6, ERBB4, NPY, PDGFD, CCL4, GCNT2, CCL3, CCL2, CCL19, NTSR2, CCR1, JUN, CCL22, TGFB1, CCL20, CFLAR, SCIMP, MT3, ESR1, EPOR, IL6, GPR183, TRPV4, FGF18, F2RL1
Positive regulation of MAPK cascade	38	1.04E-06	ITGB1, CD40, ITGB3, FLT4, TNFRSF11B, NOD2, KNG2, KNG1, LEPR, RIPK1, CD36, RELT, IL6R, NTSR2, TBX1, NGFR, IL11, GPR37, KSR1, IGFBP3, PRKCD, OSM, IGF2, LIF, GPR37L1, IGF1, TNFRSF1B, SORBS3, AGT, AR, IL6, PTPRC, ADRB3, FAS, CD27, LTBR, TNFRSF26, FGFR1
Cellular response to tumor necrosis factor	41	3.59E-06	REG3B, CCL12, CD40, CALCA, THBS1, CX3CL1, ICAM1, PYCARD, ZFP36, CCL9, ADAMTS13, MUC2, CCL7, CCL6, CCL4, ZC3H12A, CCL3, CCL2, CYP1B1, HAS2, RIPK1, CCL19, ADAMTS7, VCAM1, CCL22, NOS2, NOS3, CCL20, ERBIN, ACOD1, CYBA, MMP9, OCLN, IL6, FABP4, IRF1, GPD1, LCN2, FAS, CHI3L1, BIRC3
Integrin-mediated signaling pathway	27	6.25E-04	ITGB1, LAMA5, ITGAM, ITGB3, PLEK, ITGB2, ITGAL, PRAM1, ADAMTS2, ADAMTS1, ITGAX, ITGB8, ITGB7, ADAMTS9, FCER1G, ITGA2, ITGA1, ADAM11, VAV1, FGR, COL3A1, TYROBP, ZYX, PLP1, ITGA5, CDH17, FERMT3
Positive regulation of protein kinase B signaling	26	0.0012	CSF3, TNF, THBS1, FGF2, C1QTNF3, THPO, CCL3, CPNE1, GCNT2, CCL19, NGFR, TGFB1, MYOC, F10, IGF2, IGF1, F3, F7, IL6, MC1R, RARA, CD28, HCLS1, CHI3L1, HBEGF
Positive regulation of I-kappaB kinase/NF-kappaB signaling	38	0.0016	CD40, ECM1, EDA, HTR2B, NOD2, ZC3HAV1, TNF, LITAF, MALT1, GJA1, LGALS1, CASP8, S100A13, TNFSF10, HMOX1, FLNA, RIPK1, CD36, LGALS9, CCL19, TGM2, SECTM1B, NEK6, IRAK4, CFLAR, TICAM1, RHOC, TNFRSF1A, GPRC5B, TNIP2, CTH, IL1B, REL, F2RL1, S100A4, LTBR, MYD88, CARD11
Response to tumor necrosis factor	15	0.0020	NOS2, GCH1, MMP3, PTGS2, MMP9, CXCL16, ADAMTS13, CASP8, CHI3L1, CCL2, RIPK1, MBP, CD14, JAK2, GGT1
Intracellular signal transduction	74	0.0021	ITK, GUCY1B2, MAST3, DGKB, PLEK, HSPB1, PREX2, NRBP2, ZFP36, GRB14, CASP3, STK32A, JAK2, JAK3, GUCY1A2, CISH, PRKCD, MASTL, DGKZ, LAX1, VAV1, TIAM2, LAT2, ZAP70, DEPDC1B, MELK, TYROBP, RASA4, NRG4, SPATA13, DCX, ARHGEF2, PLCB1, SHC4, LOC100911548, GUCY2D, RGS14, SHC1, ADCY4, ASB11, NOD2, KALRN, ADCY7, RGD1562638, SOCS3, NUAK2, SOCS1, IRAK2, CHN2, CHN1, HMOX1, PLEK2, SH2B2, SOCS4, LYN, NOS2, MCF2, ECEL1, DCLK3, DCDC2, SMAD7, MOS, ADCY10, SPSB2, SPSB1, MC1R, CAMK4, STAC2, GPR182, PTPN6, PLCH2, ASB2, PLCD4, PLCD1
Tumor necrosis factor-mediated signaling pathway	16	0.0024	TNFSF18, NGFR, CD40, TNFRSF10B, TNFRSF11B, TNFRSF1B, TNF, TNFRSF1A, PYCARD, FAS, CD27, LTBR, TNFRSF26, JAK2, RELT, CARD14
Cell surface receptor signaling pathway	44	0.0024	CD63, CD274, LOC103690020, VIPR1, VIPR2, TNFRSF13B, GIPR, ITGAL, MCHR1, ADGRG3, GHRHR, UPK1B, ADGRG5, CXCR2, PRLHR, CD36, FCGR1A, CD53, LAG3, FCER1G, ANXA1, ADGRV1, TRPA1, EDN3, NPY5R, GCGR, GLP2R, TACR1, F2, TNFRSF1B, AGT, TNFRSF1A, ADGRF2, FCGR2A, MAPKAPK3, CD8A, CLCF1, TSPAN18, ADGRF4, COL4A3, CD9, OSTN, FCGR2B, MYD88
Positive regulation of tumor necrosis factor production	19	0.0039	FCER1G, CYBA, NOD2, TICAM1, NFATC4, TNFRSF1A, PYCARD, CCL4, CCL3, CCL2, RIPK1, CD36, LGALS9, CCL19, ARHGEF2, LBP, CD14, JAK2, MYD88
Positive regulation of NF-kappaB transcription factor activity	30	0.0052	CD40, EDA, ITGB2, CAMK2A, NOD2, TNF, MALT1, ICAM1, PYCARD, IRAK2, NLRP3, RIPK1, LGALS9, IL6R, TNFSF18, TGFB1, RIPK3, SPHK1, CFLAR, TICAM1, AR, IL6, CTH, IL1B, ARHGEF2, RAB7B, CARD14, MYD88, HSPA1B, CARD11
Adenylate cyclase-activating G-protein coupled receptor signaling pathway	18	0.0090	PTGFR, PTGIR, CALCA, GPR26, GCGR, PTGER2, PTGER3, ADCY4, ADRB1, HTR4, ADCY7, RXFP1, RXFP2, GHRHR, ADRB3, GALR2, UCN2, ADM2
Phospholipase C-activating G-protein coupled receptor signaling pathway	19	0.010	CASR, C5AR2, PTGER3, C5AR1, HTR2B, HTR2C, GPR84, ESR1, AGTR1A, GNG13, ANO1, CYSLTR2, GALR2, P2RY2, CXCR2, C3AR1, PLCE1, TGM2, NTSR2
Positive regulation of phosphatidylinositol 3-kinase signaling	19	0.019	CSF3, TGFB2, MYOC, IGF1, F2, AGT, FGR, SELP, GH1, ERBB4, PDGFD, RARA, CD28, F2RL1, HCLS1, PLXNB1, PTPN6, JAK2, HCST
Lipopolysaccharide-mediated signaling pathway	12	0.028	LYN, TGFB1, IRAK2, NOS3, IL1B, CCL3, CCL2, LBP, CD14, TICAM1, TNF, MYD88

**Table 4:** Alteration of biological functions related to immune and inflammatory responses in MACO model

Biological process	Number of gene	FDR	Genes
Inflammatory response	106	6.03E-23	NCF1, TRIL, TNF, NPPB, PYCARD, ZC3H12A, C3AR1, LGALS9, MAP2K3, IL4R, SLC11A1, CYBB, CYBA, NAIP6, CD8A, S100A8, PTGFR, CALCA, C5AR2, C5AR1, TNFRSF11B, MEFV, KNG2, KNG1, C3, IRAK2, NLRP3, CD14, CCR1, NGFR, SMAD1, TGFB1, TNFRSF10B, CELA1, SERPINA3N, SELE, NFKB2, SELP, CXCL10, CXCL11, IL6, CXCL12, REL, CD27, SDC1, MYD88, TP73, CXCL6, CD40, ECM1, CXCL9, SERPINA1, CXCL1, CXCL3, CXCL2, CX3CL1, CASP4, NFKBIZ, OLR1, CCR7, PTGIR, ANXA1, SPHK1, TNFRSF1B, NGF, AGTR1A, TNFRSF1A, HCK, GAL, IL1B, CHI3L1, TLR10, TNFRSF26, CD44, TLR13, CCL12, PTGER2, PTGER3, PTGS2, THBS1, PTGS1, RELB, CCL9, CCL7, CNR2, DDT, CCL6, PDPN, CCL4, SPP1, CCL3, CCL2, S1PR3, CCL19, RELT, KCNJ10, CCL22, NOS2, CCL20, KRT16, CAMK4, PLP1, FAS, F2RL1, ACKR2, LTBR
Neutrophil chemotaxis	33	4.09E-12	CCL12, CSF3R, ITGAM, C5AR1, ITGB2, CXCL1, TREM3, CXCL3, TREM1, CXCL2, CX3CL1, LGALS3, CCL9, CCL7, CCL6, CXCR2, CCL4, CCL3, SPP1, PDE4B, CCL2, CCL19, TGFB2, FCER1G, CCL22, EDN3, CCL20, ITGA1, VAV1, FCGR2A, IL1B, S100A9, S100A8
Immune response	74	3.42E-09	CXCL6, CSF3, CXCL9, EDA, LST1, CXCL1, CXCL3, TNF, CXCL2, CX3CL1, FCAR, TNFSF13B, VPREB3, ENDOU, ENPP2, TNFSF10, CCR7, CD36, ENPP3, SECTM1B, SBSPON, IL4R, RT1-CE4, RT1-CE5, TNFRSF1B, LAX1, RT1-CE3, VAV1, TNFRSF1A, ZAP70, PLSCR1, SLPI, IL1B, IRF8, TNFRSF26, RT1-CE10, C1QB, CD274, LOC103690020, CXCR5, OAS1A, RT1-A1, TNFRSF11B, RT1-A2, THBS1, OAS1F, VTN, C6, NFIL3, CCL4, RELT, CCR1, NGFR, CCL22, RT1-DMB, ZFR2, TNFSF15, TNFSF13, LIF, OSM, TNFRSF10B, PRG4, CXCL10, CXCL11, CXCL12, CD28, TNFSF9, FAS, CD27, TNFSF8, LTBR, FCGR2B, CD244, RT1-T24-4
Cellular response to interleukin-1	39	5.65E-09	CCL12, CD40, CEBPB, PTGER3, SERPINE1, HIF1A, CXCL2, CX3CL1, ICAM1, PYCARD, CCL9, CCL7, CCL6, MYC, P2RY2, CCL4, ZC3H12A, CCL3, CCL2, HAS2, APOE, CCL19, ADAMTS7, CCL22, PTGIS, NOS2, CCL20, MMP3, FN1, PAWR, ACOD1, SERPINA3N, MMP9, PSMB9, IL6, IRF1, LCN2, FAS, CHI3L1
Positive regulation of inflammatory response	29	3.37E-08	CCL12, SERPINE1, TNF, ETS1, CX3CL1, HSPD1, CCL9, EDNRA, CCL7, CCL6, CCL4, CCL3, CCL2, JAK2, LDLR, TGM2, STAT5A, TNFSF18, ACE, ITGA2, PLA2G4A, IL17RB, TNFRSF1A, GPRC5B, FABP4, TRPV4, TLR10, S100A9, S100A8
Cytokine-mediated signaling pathway	46	3.37E-08	RTN4R, LRRC15, IL1RN, CSF3R, FLT3, LRRC4, IL20RB, CSF2RB, IL2RG, CX3CL1, SOCS3, DUOX1, IL1RL2, SOCS1, LRRTM3, LRRTM1, CCL2, JAK2, JAK3, IL6R, DUOX2, SH2B2, SOCS4, STAT5A, IL4R, CISH, IL10RB, IL1R1, IL1R2, BGN, IFNLR1, IRAK4, OSMR, F3, IL17RC, IL17RB, TNFRSF1A, EREG, KLF6, IL6, IL1B, IL2RB, IL7R, IL6ST, MYD88, CD44
Response to cytokine	36	3.07E-07	CD274, OXTR, SERPINA1, SERPINE1, PTGS2, CXCL16, RELB, SOCS3, SOCS1, ACP5, TIMP1, SKIL, IL6R, TIMP4, JUN, TGFB2, NOS3, STAT3, MMP3, SERPINA3N, OSMR, SELE, NFKB2, FOSL2, FOSL1, GH1, COL3A1, IL6, MAPKAPK3, RARA, REL, FAS, IL6ST, TP53, CFTR, PTGES
Leukocyte cell–cell adhesion	16	3.10E-06	ITGB1, CALCA, VCAM1, ITGAM, ITGB2, MSN, ITGAL, SELE, ICAM1, SELP, NT5E, PTPRC, OLR1, ITGA5, EZR, FERMT3
Chemokine-mediated signaling pathway	24	8.61E-06	CCR1, CXCL6, CXCL9, CCL12, CCL22, CCL20, CXCR5, CXCR4, CXCL1, CXCL3, CXCL2, CX3CL1, CXCL10, CCL9, CXCL11, CXCL12, CCL7, CCL6, CXCR2, CCL4, CCL3, CCL2, ACKR2, CCL19
Chemotaxis	25	2.01E-05	CXCL9, C5AR2, C5AR1, HMGB2, CXCR5, CXCR4, LSP1, CX3CL1, AMOT, C3, CXCR2, CCL3, ENPP2, S1PR1, C3AR1, RAC2, PROK2, CCR7, CMKLR1, LYN, CCL20, CXCL10, CXCL11, FES, ACKR2
Acute-phase response	18	6.83E-05	REG3B, REG3A, IL1RN, SERPINA1, STAT3, HP, FN1, REG3G, F2, HIF1A, KNG1, SIGIRR, PLSCR1, IL6, TFR2, LBP, A2M, LOC100911545
Leukocyte migration	17	9.63E-05	B4GALT1, C5AR2, C5AR1, MSN, TNF, SELE, MMP9, ICAM1, SELP, SELL, PODXL, IL1B, C3AR1, PDE4B, F2RL1, ITGB7, CD34
Positive regulation of neutrophil chemotaxis	14	1.53E-04	C5AR1, CXCL1, CXCL3, CXCL2, THBS4, EDNRA, SELL, IL1B, CXCR2, C3AR1, RAC2, CCR7, CCL19, LBP
Cellular response to interferon-gamma	22	4.39E-04	CCL12, CCL22, NOS2, CCL20, ACOD1, SERPINA3N, EPRS, CX3CL1, ICAM1, CCL9, ADAMTS13, CCL7, CCL6, MYC, CCL4, CCL3, CCL2, LGALS9, CCL19, LOC685067, GBP2, GBP1
Cellular response to cytokine stimulus	18	5.96E-04	STAT5A, CD86, CSF3, MME, NOS2, FLT3, STAT3, CXCR4, MMP9, PLSCR1, IL6, IL18RAP, SOCS1, DPYSL3, HCLS1, FAS, CCR7, JAK3
Monocyte chemotaxis	15	6.93E-04	CCL12, CALCA, ANXA1, CCL22, CCL20, CX3CL1, LGALS3, CCL9, CCL7, CCL6, CCL4, CCL3, CCL2, CCL19, FOLR2
Acute inflammatory response	12	0.0020	IL6, VCAM1, B4GALT1, IL1B, HP, DEFB1, CXCL1, TACR1, TNF, S100A8, ADRA2A, PTGES
Negative regulation of inflammatory response	25	0.0029	TNFAIP6, MEFV, ETS1, C1QTNF3, SOCS3, ZFP36, NT5E, CNR2, PBK, ACP5, NLRP3, APOE, LGALS9, SLIT2, IER3, GHSR, PTGIS, ACOD1, TNFRSF1B, TNFRSF1A, SLPI, TYRO3, PTPN2, ADA, CD44
Regulation of inflammatory response	20	0.0032	LYN, ANXA1, IL1R1, NOD2, ESR1, AGT, PYCARD, IL1RL2, SBNO2, CASP12, FABP4, BCL6, DUOXA1, CASP4, ZYX, NLRP3, JAK2, GGT1, MGLL, MYD88
Cell chemotaxis	20	0.0032	CXCL6, CXCL9, CCL22, VCAM1, C5AR2, C5AR1, PRKCD, ARHGEF16, ITGA1, HMGB2, CXCL1, AGTR1A, CXCL12, CCL6, GPR183, CCL4, C3AR1, ENPP2, ENG, HBEGF
Positive regulation of leukocyte migration	11	0.0065	SELP, TNFSF18, KITLG, CCL12, ITGB3, ITGA2, CCL2, TACR1, MMP9, SELE, TP53
Lymphocyte chemotaxis	11	0.0065	CCL9, CCL12, CCL22, CCL7, CCL20, CCL6, CCL4, CCL3, CCL2, CCL19, CX3CL1
Leukocyte chemotaxis	9	0.0078	CCR1, CNR2, GPR183, CCL4, CXCR5, S1PR1, CCL3, S100A9, CXCL2
Positive regulation of phagocytosis	16	0.0088	FCER1G, SLC11A1, PROS1, CYBA, NOD2, TNF, MYH9L1, PYCARD, C3, FCGR2A, IL1B, PTX3, CD36, FCGR1A, MFGE8, FCGR2B
Positive regulation of interleukin-6 production	17	0.0090	FCER1G, CYBA, NOD2, TICAM1, TNF, HSPD1, PYCARD, IL1RL2, IL6, IL1B, TNFSF9, CD36, ARHGEF2, LBP, RAB7B, IL6R, MYD88
Receptor-mediated endocytosis	25	0.022	IFITM3, LOXL4, CXCL16, LOXL2, VTN, ENDOU, ENPP2, OLR1, APOE, ENPP3, CD14, FCGR1A, LDLR, SBSPON, MSR1, CD163, SCARA5, CD300A, PRG4, SSC5D, TMPRSS13, ADRB3, TFR2, ACKR2, FCGR2B
Positive regulation of macrophage chemotaxis	8	0.027	TNFSF18, TRPV4, C5AR1, C3AR1, CCL2, THBS1, CX3CL1, CMKLR1
Positive regulation of phagocytosis, engulfment	7	0.036	ITGA2, F2RL1, PPARG, ANO6, CD36, LBP, FCGR1A
Response to interleukin-1	14	0.038	PCSK1, IL1RN, ANXA1, IL1R1, SPHK1, MMP3, CYBA, HNMT, ETS1, SELE, IRAK2, CHI3L1, LGALS9, MYD88
Positive regulation of leukocyte chemotaxis	8	0.039	CXCL10, F7, CXCL6, CXCL11, CXCL9, EDN3, CXCR2, F2RL1

**Table 5:** Alteration of biological functions related to cell proliferation and apoptosis in MACO model

Biological process	Number of gene	FDR	Genes
Positive regulation of cell proliferation	115	3.58E-09	CSF3, TNC, SCX, RBPJ, FGF1, LEXM, ETS1, FGF2, LGALS3, EDNRA, PLAU, MYC, ENPP2, PROK2, IL6R, CASR, SOX11, POU3F2, RUNX2, EREG, AR, PGR, IL6ST, ATF3, REG3B, SHC4, PTGFR, SHMT2, HIF1A, PLAC8, GHRHR, THPO, S100A13, WNT1, LOC501901, STAT5A, LYN, CBX8, WWTR1, TGFB2, JUN, TGFB1, TNFSF13, FN1, LIF, IL31RA, IGF1, CXCL10, IL6, CXCL12, GDNF, BAMBI, CDK2, FGF18, FGFR1, ITGB1, CDCA7L, FLT4, HTR2B, KIF14, LAMC2, ADM, YBX1, GLI1, ESM1, RAC2, TIMP1, JAK2, IL11, FGFBP1, EDN3, NPY5R, SPHK1, NOG, PLA2G4A, F2, OSMR, NGF, AGTR1A, HCK, CCKBR, RARA, HCLS1, GAS1, AVP, MFGE8, HBEGF, ODC1, DOT1L, NOD2, PTGS2, HLX, ERBB4, NPY, CXCR2, S1PR1, GCNT2, HAS2, HES5, TBX1, RRM2, WNT7B, STAT3, OSM, EPOR, AGT, KITLG, FABP4, PRC1, CLCF1, MAB21L2, RGD1565660, PTPN6, FOLR2
Regulation of cell proliferation	66	1.21E-08	ITK, CXCL6, CXCL9, B4GALT1, SERPINE1, TNC, CXCL1, CXCL3, DUSP15, RBPJ, TNF, CXCL2, PLAU, CHEK1, TEAD1, APOBEC1, ANXA1, IL4R, MATK, PLA2G4A, GKN2, TNFRSF1B, TNFRSF1A, FGR, HCK, IRF1, BIRC7, TNFRSF26, TP53, LAMA5, SHC1, PTGER2, TNFRSF11B, PTGS2, HIF1A, PTGS1, CNN2, CHST11, RELT, S100A11, TCFL5, TGFB2, TFAP2C, TGFB1, JUP, NOS2, PTCH1, TNFRSF10B, CELA1, PRG4, IGF1, FA2H, CXCL10, IL6, CXCL11, BCL6, FES, TNFSF9, FAS, CD27, LTBR, MELTF, LGR5, MYD88, PLCD1, FGFR1
Positive regulation of apoptotic process	77	5.54E-06	TOP2A, ITGB1, B4GALT1, ADM, TNF, PTPRF, PYCARD, CASP8, CASP12, CASP3, TNFSF10, TSPO, CYP1B1, JAK2, PHLDA1, TGM2, ANXA1, TNFRSF12A, IGFBP3, PRKCD, PAWR, PLA2G4A, ANO6, NGF, MMP9, ALDH1A3, PLSCR1, MELK, SHQ1, FOLH1, GAL, IL1B, ALDH1A1, PPARG, TP53, GPLD1, PPP1R15A, BCL2A1, ADRB1, PTGS2, HIF1A, HSPD1, NEUROD1, LDHA, GRIN2A, C6, MUC2, ERBB4, HMOX1, PMAIP1, RIPK1, BAK1, SLIT2, MAP2K6, NGFR, TGFB2, JUN, TGFB1, ACE, NOS2, GADD45B, NOS3, GADD45A, TGFB3, OSGIN1, GADD45G, NFATC4, FOSL1, ADCY10, NELL1, REST, IL6, BCL6, FAS, MNDA, PDCD1, TP73
Organ regeneration	32	5.54E-06	CXCL6, CDKN1A, NNMT, SHC1, FLT3, C5AR1, HP, HMGB2, HTR2C, ADM, SOCS3, SOCS1, FPGS, CCL2, BAK1, UGT1A2, WNT1, ANGPT2, TGFB1, ACE, ANXA3, LIF, APOA5, HSPG2, F7, CXCL12, NR4A3, CDK2, CDK1, PPARG, ALDOC, LCP1
Positive regulation of smooth muscle cell proliferation	28	8.68E-05	SHC1, ITGB3, PTGS2, RBPJ, TNF, FGF2, HIF1A, CX3CL1, MYC, PDGFD, S1PR1, C3AR1, HMOX1, IL6R, HES5, TGM2, JUN, NPY5R, ITGA2, CYBA, IRAK4, IGF1, SULF1, EREG, IL6, NR4A3, MYD88, HBEGF
Negative regulation of cell proliferation	78	1.26E-04	ITGB1, IFITM3, CDKN1A, B4GALT1, TES, FLT3, ADM, RBPJ, TNF, FGF2, PTPRF, RXFP2, NPPC, GJA1, BDKRB2, WDR6, CYP1B1, JAK2, SOX7, TGIF1, IGFBP3, ITGA1, P3H2, WNT9A, ETV3, HSPG2, EREG, RUNX1, AR, GAL, IL1B, IRF1, RARA, PPARG, TAX1BP3, TP53, GPLD1, PTGES, PPP1R15A, SLFN2, TWIST2, PTGS2, DLL4, CYP27B1, MUC2, ERBB4, XIRP1, HMOX1, BAK1, SLIT2, LYN, BCHE, SMAD1, TGFB2, JUN, TGFB1, NOS3, VDR, TGFB3, PTCH1, STAT3, LIF, HMGA1, OSM, FUZ, INHBA, IGF1, SMARCA2, AGT, FOSL1, REST, IL6, BCL6, CTH, GJB6, CD9, PTPN2, TP73
Positive regulation of fibroblast proliferation	23	0.0012	TGIF1, NGFR, CDKN1A, JUN, TGFB1, ANXA2, SPHK1, ITGB3, SERPINE1, FNDC3B, FN1, IGF1, ESR1, KNG2, KNG1, AGT, FOSL2, AQP1, EREG, ZMIZ1, MYC, PDGFD, WNT1
Brown fat cell differentiation	15	0.0014	CEBPB, LAMB3, EBF2, ADRB1, PTGS2, PLAC8, SELENBP1, RGS2, ALDH6A1, FABP4, LRG1, ADRB3, MB, PPARG, SH2B2
Endodermal cell differentiation	13	0.0030	LAMB3, ITGB2, LAMA3, FN1, INHBA, MMP8, MMP9, VTN, MMP14, MMP15, COL7A1, COL8A1, ITGA5
Apoptotic process	71	0.0030	CLIC4, NCF1, BUB1B, FAIM2, ECE1, HIF3A, AHR, TNF, LITAF, PYCARD, CASP7, GJA1, CASP8, LGALS1, CASP12, PPP1R13L, CASP3, ZC3H12A, CASP4, PIM1, TRIM69, MAP3K8, PHLDA1, LGALS7, CTSC, CASR, DCC, PRKCD, PAWR, DIO3, TOX3, PLSCR1, MELK, CCKBR, IRF1, CHI3L1, ALDOC, AVP, DNASE1, TP53, S100A9, S100A8, BIRC3, SHC4, PPP1R15A, ASAH2, BCL2A1, SEMA3A, C5AR1, SLC5A11, ADRB1, NUAK2, MUC2, CCL6, PMAIP1, RIPK1, BAK1, MAP2K6, SIAH3, RHBDD1, NEK6, CFLAR, MT3, CIDEC, GADD45G, GZMM, GJB6, CDK1, FAS, FGF13, XAF1
Negative regulation of apoptotic process	91	0.0083	CDKN1A, IL1RN, FLT4, HHIP, HTR2B, KIF14, SCX, HSPB1, SLC2A3, YBX1, RXFP2, AQP1, LGALS3, EDNRA, CASP3, CLEC5A, PIM1, PROK2, SOX8, TIMP1, JAK2, IER3, SERPINB2, NPY5R, SPHK1, PLAUR, NGF, MMP9, TNFRSF1A, ADAMTS20, HCK, RARA, GAS1, PGR, BIRC7, AVP, ANGPTL4, TP53, CARD14, CRYAB, STK40, CD44, BIRC3, REG3B, PTGFR, LTK, BCL2A1, TWIST2, THY1, THBS1, HIF1A, HSPD1, PLAC8, SOCS3, NUAK2, CHST11, IRAK2, CBS, ERBB4, FIGNL1, CXCR2, FLNA, LOC501901, STAT5A, NGFR, TGFB2, JUN, RHBDD1, STAT3, FN1, CFLAR, IGF1, MT3, KITLG, IL6, NR4A3, LHX3, BCL6, CTH, IL2RB, BCL3, ALB, CDK1, FAS, CD27, RGD1565660, PDCD1, MYD88, ADA, HSPA1B, LIMS1
Apoptotic signaling pathway	17	0.025	NGFR, CD40, PTGIS, VDR, PAWR, TNFRSF11B, TICAM1, NGF, TNFRSF1B, TNF, CASP8, CD28, FAS, LTBR, TNFRSF26, BAK1, RELT

**Table 6:** Alteration of biological functions related to neurological disorders in MACO model

Biological process	Number of gene	FDR	Genes
Aging	84	1.43E-09	TOP2A, CD86, NCF2, ELN, ITGB2, NPY2R, HP, HSPB1, ADM, LITAF, FGF2, CX3CL1, EDNRA, CASP7, CASP12, KRT25, LEPR, TSPO, TIMP1, GGT1, CTSC, SREBF1, NPY5R, IGFBP3, PRKCD, NGB, PLA2G4A, TACR3, GFRA1, NGF, TNFRSF1B, MMP9, F3, AGTR1A, ADRB3, DPYD, SERPING1, ALDOC, TP53, CRYAB, PPP1R15A, C1QB, CALCA, BCL2A1, CNP, SHC1, ADRB1, AURKB, PTGS1, CYP27B1, SOCS3, NPY, P2RY2, CCL2, DMD, MBP, APOE, BAK1, LDLR, TGFB2, JUN, VCAM1, TGFB1, ACE, NOS2, MOG, NOS3, VDR, TGFB3, STAT3, IGF1, CP, EPOR, AGT, NFKB2, FOSL2, IL6, COL3A1, KRT16, GJB6, KRT14, FAS, VIM, ADA
Response to axon injury	17	0.0059	LYN, PCSK1, GSTM1, MUSK, GIPR, NOS3, TXNRD1, LTC4S, FGF2, UCK2, LGALS1, NAIP6, DPYSL3, CDK1, TSPO, MATN4, MATN2
Stabilization of membrane potential	9	0.012	KCNK5, LOC100909725, KCNK6, KCNK10, KCNK13, KCNK15, KCNN4, KCNK1, KCNK4
Neuron differentiation	34	0.036	RET, BARHL2, CEBPB, WNT2B, SHC1, BHLHE22, RBPJ, HDAC9, NRBP2, WNT6, CASP3, S1PR1, WNT1, RXRG, HES5, HELT, WNT5B, EDN3, CNTN6, WNT7B, WNT9B, EN1, SOX11, TBR1, GFRA1, WNT9A, POU3F2, RUNX2, RUNX1, VGF, GDNF, LHX2, NES, HSPA1B
Myeloid dendritic cell differentiation	10	0.037	CD86, BATF3, SPI1, TGFB1, CAMK4, TNFSF9, LTBR, RBPJ, BATF, RELB
Mature conventional dendritic cell differentiation	5	0.039	F2RL1, PRTN3, CCR7, LGALS9, CCL19
Memory	23	0.042	IL1RN, CEBPB, OXTR, KCNK10, MUSK, NOG, IGF2, ADRB1, IGF1, NGF, PTGS2, SORCS3, KALRN, SLC6A4, PTGS1, SLC8A2, GRIN2A, JPH3, IL1B, ITGA5, FGF13, PLCB1, KCNK4
Negative regulation of neuron apoptotic process	35	0.022	REG3B, GABRB2, CEBPB, SCT, C5AR1, KIF14, FAIM2, GRIK2, HIF1A, HSPD1, NRBP2, CCL2, HMOX1, APOE, JAK2, NGFR, JUN, GABRA5, TGFB3, FZD9, EN1, MT1, NGF, MT3, EPOR, AGT, TOX3, NR4A3, GDNF, CLCF1, GFRAL, TYRO3, NES, TP73, LGMN

**Fig. 3:**
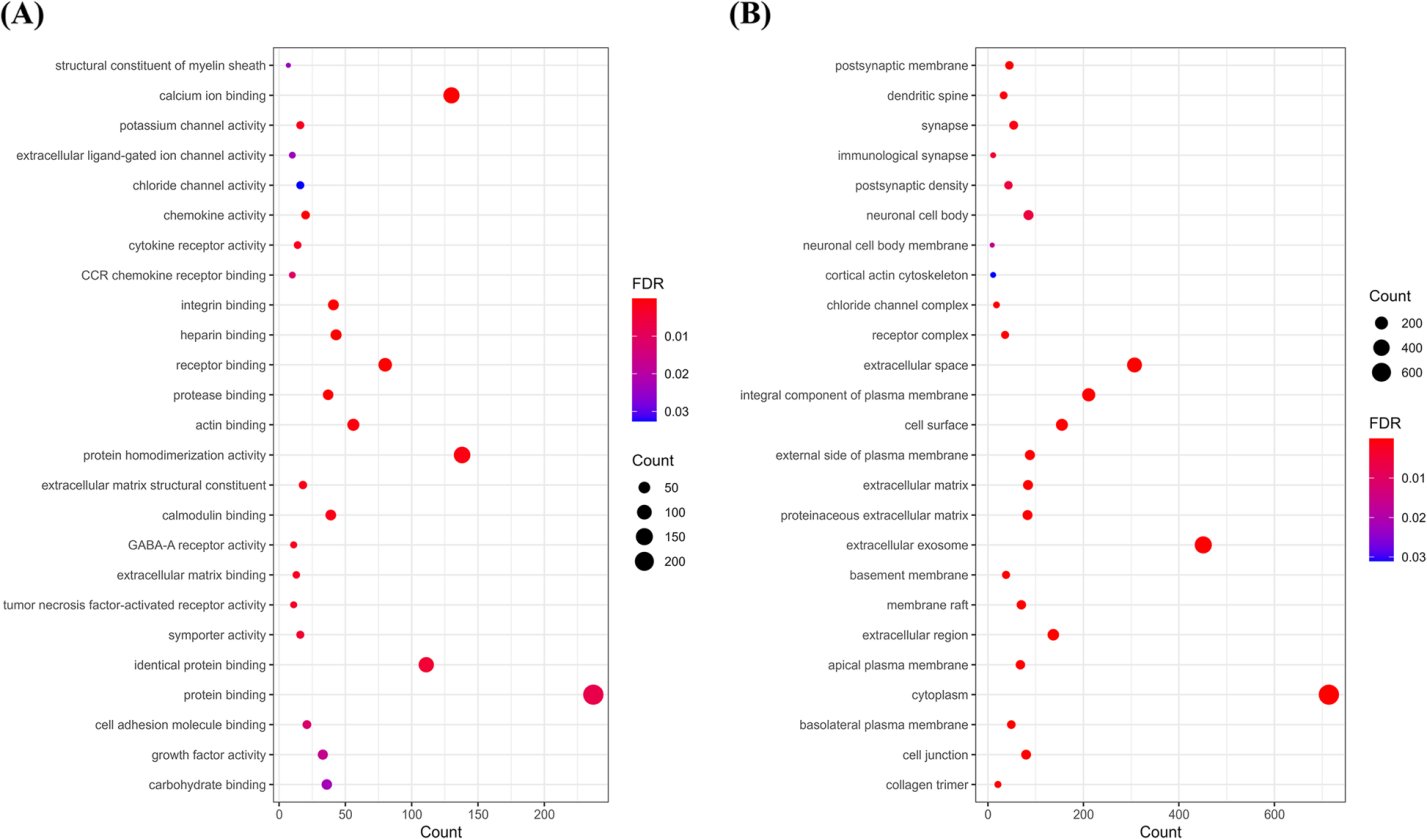
Differentially expressed genes are involved in ion channel activities and synapse functions in the middle cerebral artery occlusion model. Gene ontology enrichment analysis highlighted the involvement of the differently expressed genes in **A** the molecular functions of ion channel activities and receptor binding and **B** cellular components of neuronal cells. The size of the dots represents the number of genes, and the color of the dots represents the significance of the terms

In the GO analysis of molecular function, we also found the structural alteration of myelin sheath and alteration of many functions related to ion channels such as calcium ion binding, potassium channel activity, extracellular ligand-gated ion channel activity, and chloride channel activity (Fig. 3A) (*p* < 0.05). Additionally, immune and inflammatory-related activity, such as chemokine activity, cytokine receptor activity, and C–C motif chemokine receptor binding, was highlighted in our results. In the GO analysis of cellular components, we observed the involvement of postsynaptic membrane, dendritic spine, synapse, immunological synapse, postsynaptic density, neuronal cell body, neuronal cell body membrane, and cortical actin cytoskeleton (Fig. 3B) (*p* < 0.05). Taken together, our data suggested the contribution of the dysregulated gene in the pathology of the stroke model.

### Alteration of gene network involved in brain disorders in the MCAO model

**Fig. 4:**
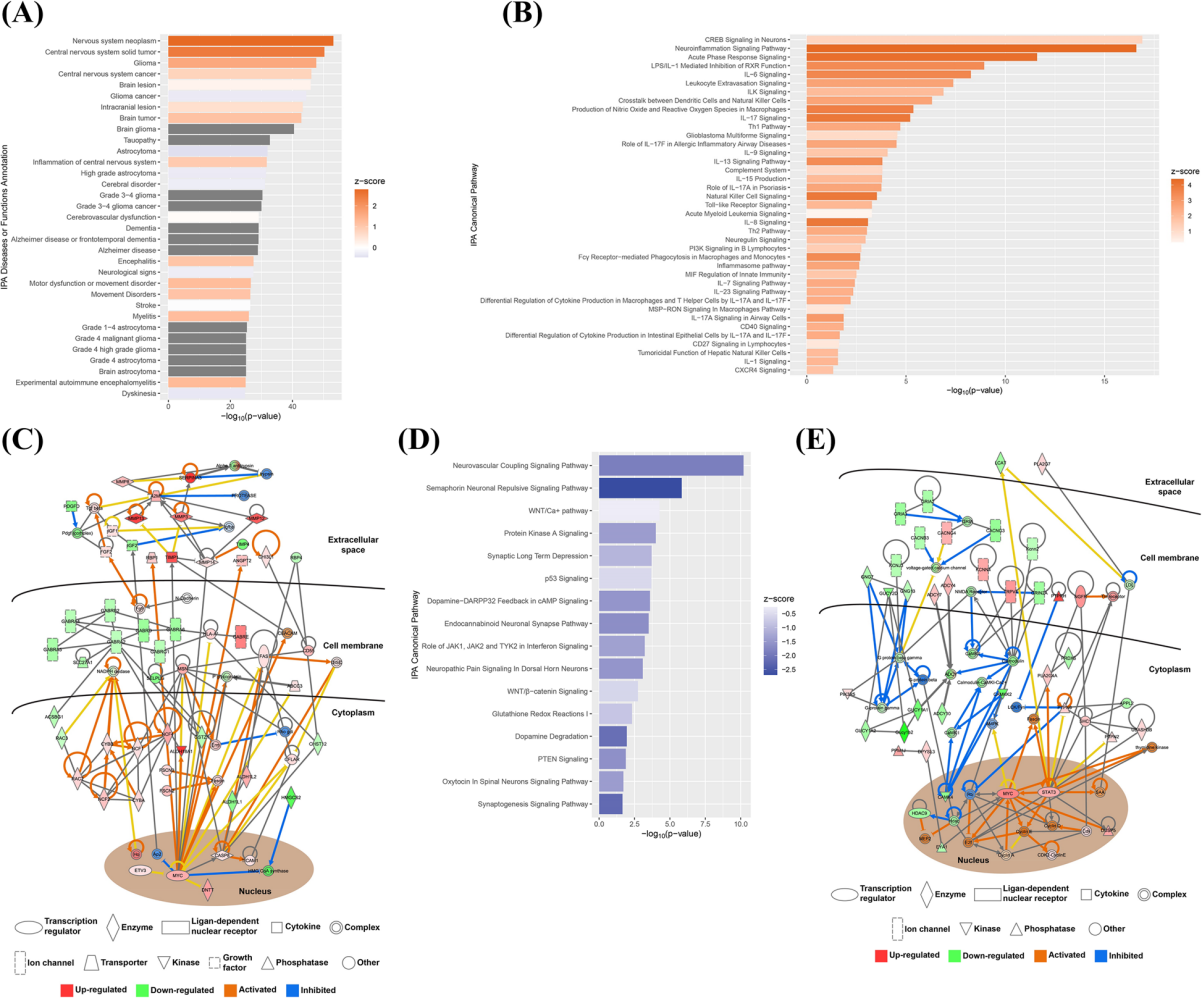
Alterations of cell signaling pathways in the middle cerebral artery occlusion (MCAO) model. **A** Significant change in neural biological functions and diseases controlled by the dysregulated genes in the MCAO model. Color of Z-score represents the degree of activation or inhibition of the functions and diseases. **B** Activation of canonical pathways related to neural functions and inflammatory and immune responses. The color of the Z-score represents the degree of activation. **C** The gene network construction revealed the involvement of different ion channels, enzymes, receptors, transcription factors, and kinases in the activation of inflammatory and immune responses in the MCAO model. Red squares represent upregulated genes, green squares represent downregulated genes, orange arrows represent activated pathways, and blue arrows represent inhibited pathways. **D** Inhibition of canonical pathways related to neural functions. The color of the Z-score represents the degree of inhibition. **E** The Ingenuity Pathway Analysis demonstrated the contribution of different ion channels, enzymes, receptors, transcription factors, and kinases in the inhibition of neural functions in the MCAO model. Red squares represent upregulated genes, green squares represent downregulated genes, orange arrows represent activated pathways, and blue arrows represent inhibited pathways

To delineate the molecular mechanisms and gene network involved in brain disorders in the MCAO model, IPA was conducted. In the diseases and biological functions of IPA, our results highlighted many neurological diseases and abnormalities, especially cerebrovascular dysfunction, and stroke (Fig. 4A) (*p* < 0.05). There were 107 DEGs that were closely associated with stroke (Table 7) (q < 0.05). Moreover, we further looked at the altered canonical pathways in the stroke model. We found that activation of cell signaling was related to immune and inflammatory response pathways such as the neuroinflammation signaling pathway, inflammasome pathway, integrin-linked kinase (ILK) signaling, and Th1 pathway (Fig. 4B) (*p* < 0.05). Additionally, neuronal functions-and disorders-related signaling pathways, including cAMP response element-binding protein signaling in neurons, glioblastoma multiform signaling, and neuregulin signaling, were also highlighted (Fig. 4B) (*p* < 0.05). The network analysis showed the involvement of many ion channels, enzymes, cytokines, and complexes in these activations (Fig. 4C) (*p* < 0.05). On the other hand, the IPA analysis showed the inhibition of signaling was related to brain functions such as neurovascular coupling signaling pathway, semaphorin neuronal repulsive signaling pathway, endocannabinoid neuronal synapse pathway, dopamine-DARPP32 feedback in cAMP signaling, dopamine degradation, oxytocin in spinal neurons signaling pathway (Fig. 4D) (*p* < 0.05). Moreover, some important cell signaling pathways, including WNT/Ca^+^, protein A signaling, p53 signaling, Janus kinases (JAK1 and JAK2), tyrosine kinase 2 in interferon signaling, WNT/Î^2^-catenin signaling, and phosphatase and tensin homolog signaling, were also found to be inhibited in the stroke model. The gene network construction further demonstrated the contribution of ion channels, enzymes, and transcriptional factors in controlling these inhibitions (Fig. 4E) (*p* < 0.05).

**Table 7:** DEGs associated with stoke

Gene name	Gene symbol
Angiotensin I converting enzyme	ACE
Adenosine deaminase	ADA
Adrenoceptor alpha 2A	ADRA2A
Adrenoceptor alpha 2C	ADRA2C
Adrenoceptor beta 1	ADRB1
Adrenoceptor beta 3	ADRB3
Angiotensinogen	AGT
Angiotensin II receptor type 1	AGTR1
Albumin	ALB
Apolipoprotein E	APOE
Androgen receptor	AR
ATPase Na + /K + transporting subunit alpha 1	ATP1A1
ATPase Na + /K + transporting subunit alpha 2	ATP1A2
Butyrylcholinesterase	BCHE
B-cell CLL/lymphoma 6	BCL6
Carbonic anhydrase 13	CA13
Carbonic anhydrase 14	CA14
Carbonic anhydrase 2	CA2
Carbonic anhydrase 3	CA3
Carbonic anhydrase 9	CA9
Calcium voltage-gated channel auxiliary subunit beta 3	CACNB3
CD36 molecule	CD36
Cholinergic receptor muscarinic 1	CHRM1
Cholinergic receptor muscarinic 3	CHRM3
Cholinergic receptor muscarinic 4	CHRM4
Cholinergic receptor nicotinic beta 3 subunit	CHRNB3
Cholinergic receptor nicotinic beta 4 subunit	CHRNB4
Collagen type IV alpha 1 chain	COL4A1
C-X-C motif chemokine ligand 16	CXCL16
Cytochrome b-245 beta chain	CYBB
Dopa decarboxylase	DDC
Dopamine receptor D4	DRD4
Endoglin	ENG
Estrogen receptor 1	ESR1
Coagulation factor X	F10
Coagulation factor II	F2
Coagulation factor V	F5
Fas cell surface death receptor	FAS
Fibronectin 1	FN1
Gamma-aminobutyric acid type A receptor alpha2 subunit	GABRA2
Gamma-aminobutyric acid type A receptor alpha4 subunit	GABRA4
Gamma-aminobutyric acid type A receptor alpha 5 subunit	GABRA5
Gamma-aminobutyric acid type A receptor alpha 6 subunit	GABRA6
Gamma-aminobutyric acid type A receptor beta 2 subunit	GABRB2
Gamma-aminobutyric acid type A receptor delta subunit	GABRD
Gamma-aminobutyric acid type A receptor epsilon subunit	GABRE
Gamma-aminobutyric acid type A receptor gamma 1 subunit	GABRG1
Gamma-aminobutyric acid type A receptor pi subunit	GABRP
Gamma-aminobutyric acid type A receptor theta subunit	GABRQ
Gamma-aminobutyric acid type A receptor rho 1 subunit	GABRR1
Gap junction protein	GJA1
Glycine receptor	GLRA1
Glutamate ionotropic receptor NMDA type subunit 2A	GRIN2A
Glutamate ionotropic receptor NMDA type subunit 2C	GRIN2C
Hepatocarcinoma resistance QTL 2	HCAR2
Histone deacetylase 9	HDAC9
Heat shock protein family B	HSPB1
5-hydroxytryptamine receptor 2B	HTR2B
5-hydroxytryptamine receptor 2C	HTR2C
Insulin-like growth factor 1	IGF1
Interleukin 1 receptor type 1	IL1R1
Integrin subunit alpha M	ITGAM
Integrin subunit beta 3	ITGB3
Integrin subunit beta 8	ITGB8
Potassium voltage-gated channel subfamily H member 7	KCNH7
Lipocalin 2	LCN2
Maltase-glucoamylase	MGAM
Matrix metallopeptidase 9	MMP9
Nerve growth factor receptor	NGFR
Nicotinamide N-methyltransferase	NNMT
Nitric oxide synthase 2	NOS2
Nitric oxide synthase 3	NOS3
Neuropeptide Y	NPY
Oxidized low density lipoprotein	OLR1
Purinergic receptor P2Y12	P2RY12
Phosphodiesterase 4B	PDE4B
Phosphodiesterase 4C	PDE4C
Progesterone receptor	PGR
Phospholipase A2 group VII	PLA2G7
Plasminogen activator	PLAT
Peroxisome proliferator-activated receptor gamma	PPARG
Protein C receptor	PROCR
Protein S	PROS1
Proteinase 3	PRTN3
Prostaglandin E synthase	PTGES
Prostaglandin-endoperoxide synthase 1	PTGS1
Prostaglandin-endoperoxide synthase 2	PTGS2
Phosphorylase	PYGL
S100 calcium binding protein A9	S100A9
Sodium channel epithelial 1 alpha subunit	SCNN1A
Serpin family A member 1	SERPINA1
Serpin family D member 1	SERPIND1
Serpin family E member 1	SERPINE1
Solute carrier family 1 member 2	SLC1A2
Solute carrier family 2 member 1	SLC2A1
Solute carrier family 6 member 4	SLC6A4
Thrombospondin 1	THBS1
Tumor necrosis factor	TNF
TNF receptor superfamily member 12A	TNFRSF12A
TNF receptor superfamily member 1A	TNFRSF1A
Troponin T2	TNNT2
Tumor protein p53	TP53
Translocator protein	TSPO
Tubulin	TUBA1C
Tubulin	TUBA4A
Vascular cell adhesion molecule 1	VCAM1
Visinin-like 1	VSNL1

### Validation of transcriptome finding

**Fig. 5:**
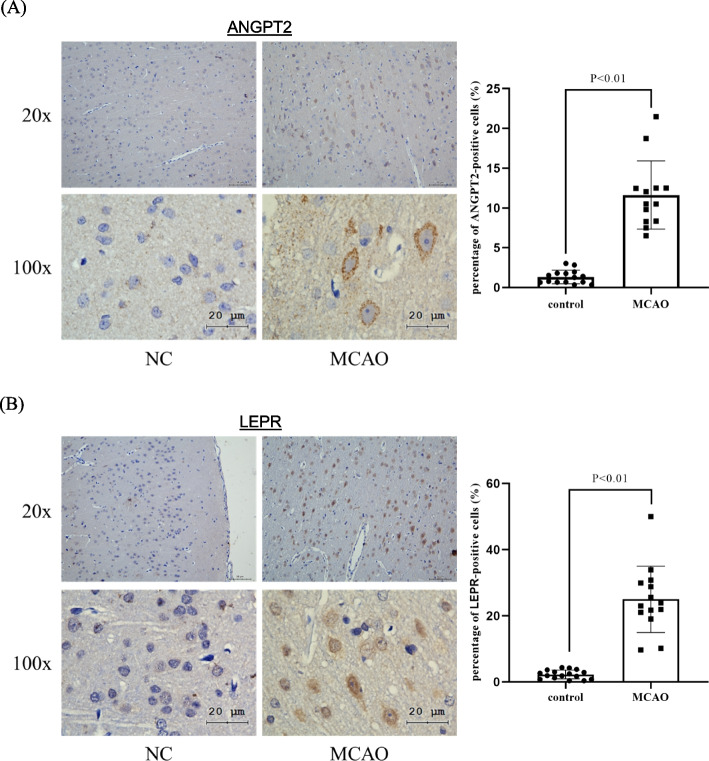
Validation of the findings from transcriptome sequencing. Immunohistochemistry (IHC) staining showed the induction of **A** Angpt2 and **B** Lepr in the brain tissue section of MCAO model. Left panel was the representative image of IHC staining. Right panel was the graph that showed the % of positive stained cells

To validate the findings from transcriptome sequencing, immunohistochemistry staining was used to evaluate the induction of the 2 identified biomarkers of ischemia stroke. The results of IHC well-matched with the findings of transcriptomic analysis that both Angpt2 and Lepr were induced in MACO model (Fig. 5A & B) (*p* < 0.05).

## Discussion

In the present study, we aimed to delineate the molecular mechanisms underlying the development of ischemic stroke. Based on our findings, we identified novel biomarkers for early detection and therapy of ischemic stroke. By using the well-established MCAO model and comparative transcriptome sequencing, we identified over 2000 DEGs in the brain tissue of the model [13]. The GO analysis on the DEGs highlighted their importance in many biological processes that contributed to the development of ischemic stroke. In the analysis, we mainly focused on the biological processes related to oxygen regulation. Our result showed a cluster of genes that played important roles in angiogenesis and hypoxia response. These can be potential biomarkers for early detection of ischemic stroke or therapeutic targets for ischemic stroke treatment. Some of our findings matched previous reports. For instance, we found the induction of heme oxygenase-1 (HMOX1) in the brain tissue of the MCAO model, which is concordant to the results in a similar study that used a middle cerebral artery embolization reperfusion rat model [14]. HMOX1 gene is responsible for encoding the enzyme heme oxygenase 1, which is a stress-induced enzyme that plays role in response to oxygen depletion [15]. Moreover, the overexpression of HMOX1 is commonly associated with neurodegenerative diseases, including ischemic stroke [16]. Clinically, a randomized controlled trial study on 30 chronic stroke patients suggested that serum HMOX1 levels could be used as a biomarker of stroke [17]. Furthermore, our results showed that there was an induction of hypoxia-inducible factor 1 subunit alpha (HIF1α), which is a well-known responder of low oxygen levels in the brain [18]. It was reported that HIF1α could regulate the progress of neurological symptoms after ischemic stroke [19]. Furthermore, a focal cerebral ischemic rat study showed that HIF1α plays a role to protect the vascular structure and promote angiogenesis [20].

In addition, we also observed the overexpression of an angiopoietin gene family, including angiopoietin-like protein 4 (Angptl4) and Angpt2. Angptl4 is already reported as a prognostic marker for ischemic stroke because higher levels of Angptl4 in the plasma were associated with poor prognosis in acute ischemic stroke patients and were a predictive biomarker in atherosclerosis [21, 22]. Many clinical studies have suggested using Angptl4 for post-stroke treatment because it could enhance angiogenesis and neurogenesis by reducing neuronal death and inflammatory response [23]. In addition, Angptl4 showed a neuroprotective effect in the treatment of cerebral ischemia [24]. Mouse studies further suggested that Angptl4 counteracted the loss of vascular integrity [25] and protected the permeability of the blood–brain barrier damaged by ischemic stroke [26]. On the other hand, our result suggested that Angpt2 could be a novel biomarker for the early detection of ischemic stroke. The elevation of Angpt2 that was concordant to a mouse study that reduced inspired oxygen could increase expression of Angpt2 [27]. Angpt2, an angiogenetic marker, was found to be associated with destabilized endothelial cell junctions and enlarged lumen formation [28]. Angpt2 was considered as a proangiogenic factor that could be responsible for endothelial dysfunction and increased risk for vascular disorders [29] and functioned as a biomarker to predict the life expectancy of peripheral arterial disease patients [30]. A multi-center case–control study on stroke patients further showed the association of variants in the promoter of Angpt2 with stroke [31]. A knockout mice study also demonstrated that treatment of Angpt2-neutralizing antibody attenuated the defects in vascular malformations, which shows that Angpt2 can be used for treatment and is not only important as a biomarker [28]. Other mice studies also suggested that Angpt2 was an important mediator of arteriovenous malformations through the control of TGFβ signaling [32]. Moreover, we showed the overexpression of Lepr in the brain of the MCAO model. Lepr is a type I cytokine receptor that functions as a receptor for the fat cell-specific hormone leptin [33]. A study using Leprdb/db diabetic mouse model demonstrated the association of diabetes mellitus with central nervous system pathologies, including stroke [34]. A meta-analysis conducted by Wu’s group suggested that LEPR polymorphism was significantly associated with the increased risk of cardiovascular disease [35]. Another study of nucleotide polymorphisms in 101 patients with ischemic stroke and 105 controls also showed the association of Lepr polymorphisms and increased risk of ischemic stroke [36], suggesting the potential use of Lepr as a genetic predictive factor for ischemic stroke.

Other than the identification of biomarkers for early detection of ischemic stroke, we also aimed to delineate the molecular mechanisms underlying the development of stroke. We applied IPA, an advanced bioinformatic tool, to analyze the comparative transcriptome data. In this analysis, we primarily focused on the canonical pathways associated with inflammatory responses and neurological dysfunctions in the stroke model. Our results highlighted the activation of the inflammasome pathway, ILK signaling, and the Th1 pathway. Inflammasome, a cytosolic multiprotein complex, plays important roles in the innate immune system in response to inflammation [37]. It controls the activation of caspase-1, which cleaves pro-inflammatory cytokines, including pro-IL-1 and pro-IL-18 [37]. It was reported that inflammasome from microglial cells play an inflammatory role in the hypoxic-ischemic encephalopathy model [38]. A clinical study suggested that inflammasome-related inflammatory factor IL-1β is correlative with cerebral small vessel disease patients [39]. A rat study also demonstrated that the inhibition of inflammasome activity could alleviate inflammatory injury in the MCAO model [40, 41]. Hence, the therapeutic target of the inflammasome may provide a novel approach to treat ischemic stroke. In our study, we also observed the activation of the ILK signaling pathway and Th1 signaling pathway in the brain tissue of the MCAO model. ILK signaling pathway contributes to the regulation of cellular adhesion and cell apoptosis under cell death stimuli [42]. In addition, ILK signaling was reported to regulate VEGF expression through the IL-6 pathway [43]. The ILK pathway was found to be dysregulated in neurodegeneration after traumatic brain injury [44]. More importantly, the ILK pathway was reported to regulate apoptotic cell death after focal cerebral ischemia [42]. Other than the ILK signaling, we also observed the alteration of the Th1 pathway in the MCAO model. Th1 signaling is responsible for perpetuating autoimmune responses [45]. It was found that Th1 signaling is dysregulated in neuroinflammation and CNS autoimmune diseases [46]. In addition, Th1 signaling is reported to promote the development of cerebral ischemic stroke [47]. Furthermore, an autoimmune myocarditis rat model demonstrated that inhibition of Th1 inflammatory cytokines could attenuate the autoimmune myocarditis [48]. Hence, targeting the ILK and Th1 pathways might be a potentially efficacious clinical approach for treating ischemic stroke.

In conclusion, our study discovered novel biomarkers, such as Angpt2 and Lepr, that could be used for the early detection of ischemic stroke. Moreover, we provided insight into the molecular mechanisms underlying the inflammatory responses and alteration of neural functions in the MCAO model. Our results provide possible pharmaceutical targets for treating ischemic stroke. However, further pre-clinical studies are necessary to warrant the findings of the present works. For instance, a comparative analysis on the serum or cerebrospinal fluid samples from clinical stroke patients would provide us a better understanding on the possible use of these markers for the early detection of ischemic stroke.

## Supplementary Information

**Additional file 1.**

## Data Availability

Raw reads of the transcriptomes have been deposited in NCBI's BioProject under the accession number PRJNA800260.
